# 6-Hy­droxy-5,7,8-trimethyl­chroman-2-one

**DOI:** 10.1107/S1600536811019982

**Published:** 2011-06-04

**Authors:** Shailesh K. Goswami, Lyall R. Hanton, C. John McAdam, Stephen C. Moratti, Jim Simpson

**Affiliations:** a Department of Chemistry, University of Otago, PO Box 56, Dunedin, New Zealand

## Abstract

The title compound, C_12_H_14_O_3_, consists of a chromanone unit with an –OH substituent at the 4-position and methyl substituents on the remaining C atoms of the aromatic ring. The fused pyran­one ring adopts a distorted envelope conformation with the methyl­ene group adjacent to the carbonyl carbon as the flap atom. The crystal structure is stabilized by classical O—H⋯O hydrogen bonds and weak C—H⋯O and C—H⋯π inter­actions, generating a three-dimensional network.

## Related literature

For the synthesis, see: Ong *et al.* (2008 ▶). For a related structure, see: Budzianowski & Katrusiak (2002 ▶). For current applications of this compound, see: Ong *et al.* (2008 ▶); Harada *et al.* (1987 ▶); Hernández-Torres *et al.* (2009 ▶). For bond-length data, see: Allen *et al.* (1987 ▶).
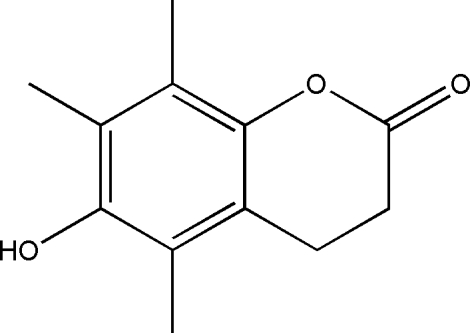


## Experimental

### 

#### Crystal data

C_12_H_14_O_3_
*M* *_r_* = 206.23
Monoclinic, 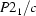

*a* = 4.5339 (6) Å
*b* = 16.815 (2) Å
*c* = 13.302 (2) Å
β = 96.495 (8)°
*V* = 1007.6 (2) Å^3^
*Z* = 4
Mo *K*α radiation
μ = 0.10 mm^−1^
*T* = 89 K
0.38 × 0.11 × 0.06 mm

#### Data collection

Bruker APEXII CCD area-detector diffractometer
Absorption correction: multi-scan (*SADABS*; Bruker 2009 ▶) *T*_min_ = 0.809, *T*_max_ = 1.00
12531 measured reflections
2021 independent reflections
1535 reflections with *I* > 2σ(*I*)
*R*_int_ = 0.063

#### Refinement

*R*[*F* ^2^ > 2σ(*F* ^2^)] = 0.070
*wR*(*F* ^2^) = 0.201
*S* = 1.13
2021 reflections
142 parameters
1 restraint
H atoms treated by a mixture of independent and constrained refinement
Δρ_max_ = 0.39 e Å^−3^
Δρ_min_ = −0.27 e Å^−3^

### 

Data collection: *APEX2* (Bruker 2009 ▶); cell refinement: *SAINT* (Bruker 2009 ▶); data reduction: *SAINT*; program(s) used to solve structure: *OLEX2* (Dolomanov *et al.*, 2009 ▶); program(s) used to refine structure: *SHELXL97* (Sheldrick, 2008 ▶) and *TITAN2000* (Hunter & Simpson, 1999 ▶); molecular graphics: *SHELXTL* (Sheldrick, 2008 ▶) and *Mercury* (Macrae *et al.*, 2008 ▶); software used to prepare material for publication: *SHELXL97*, *enCIFer* (Allen *et al.*, 2004 ▶), *PLATON* (Spek, 2009 ▶) and *publCIF* (Westrip, 2010 ▶).

## Supplementary Material

Crystal structure: contains datablock(s) I, global. DOI: 10.1107/S1600536811019982/hg5043sup1.cif

Structure factors: contains datablock(s) I. DOI: 10.1107/S1600536811019982/hg5043Isup2.hkl

Supplementary material file. DOI: 10.1107/S1600536811019982/hg5043Isup3.cml

Additional supplementary materials:  crystallographic information; 3D view; checkCIF report

## Figures and Tables

**Table 1:** Hydrogen-bond geometry (Å, °) *Cg*2 is the centroid of the C1–C6 benzene ring.

*D*—H⋯*A*	*D*—H	H⋯*A*	*D*⋯*A*	*D*—H⋯*A*
O4—H4*O*⋯O9^i^	0.85 (2)	2.02 (3)	2.754 (3)	144 (3)
C8—H8*B*⋯O9^ii^	0.99	2.58	3.395 (4)	140
C8—H8*B*⋯O4^iii^	0.99	2.66	3.440 (4)	136
C7—H7*B*⋯*Cg*2^iv^	0.99	2.61	3.505 (3)	150
C31—H31*C*⋯*Cg*2^v^	0.98	2.62	3.512 (3)	151

Symmetry codes: (i) 
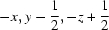
; (ii) 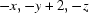; (iii) 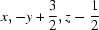; (iv) 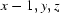; (v) 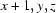.

**Fig. 1.**
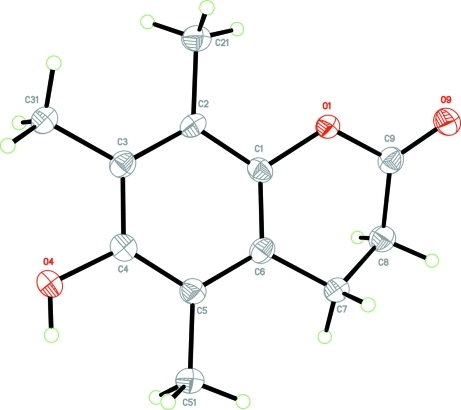
The structure of (I) showing the atom numbering with ellipsoids drawn at the 50% probability level.

**Fig. 2.**
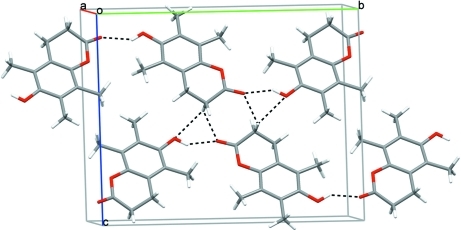
bc layer of (I). Dashed lines show O–H···O hydrogen bonds and C–H···O interactions.

**Fig. 3.**
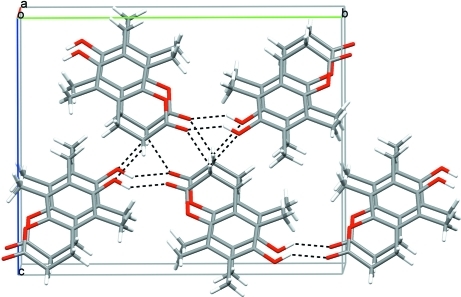
Crystal packing of (I) showing the three dimensional network structure. Hydrogen bonds are drawn as dashed lines.

## supplementary crystallographic information

### Comment

The title compound (I) has been utilized in the synthesis of important members of the Vitamin E family (Harada *et al.*, 1987; Hernández-Torres *et al.*, 2009) and as a redox-trigger in liposome research (Ong *et al.*, 2008). We have utilized (I) in synthesis of redox-active quinone monomers as part of our current interest in electro-mechanical actuators.

The structure of (I), Fig. 1, consists of a chromanone unit with an OH substituent at the 4-position and methyl substituents on C2, C3 and C5. The fused pyranone ring adopts a distorted envelope conformation with the C8 atom as the flap atom. Bond distances (Allen *et al.*, 1987) and angles are normal and similar to those in the closely related compound with two methyl substituents at C7 (4,4-dimethyl-6-hydroxy-5,7,8-trimethylchroman-2-one) (Budzianowski & Katrusiak, 2002).

In the crystal structure classical O4–H4O···O9 hydrogen bonds form zigzag chains down the *b* axis. Weaker C8–H8B···O4 and C8–H8B···O9 interactions link the chains into sheets in the *bc* plane (Fig. 2). The structure is further stabilized by C7–H7B···π and C31–H31C···π interactions forming stacks down *a*, Fig 3.

### Experimental

The title compound was prepared (Ong *et al.*, 2008) by a Friedel-Crafts type addition reaction of trimethylhydroquinone with acrylic acid using methanesulfonic acid as the acid catalyst in dichloroethane at 100°C for 2 h. X-ray quality crystals were grown from aqueous ethanol.

### Refinement

The OH hydrogen atom was located in a difference Fourier map and refined with the O–H distance restrained to 0.85 (2) Å and *U*_iso_ = 1.2*U*_eq_ (O). Methyl and methylene H-atoms were refined using a riding model with d(C—H) = 0.98 Å, *U*_iso_=1.5*U*_eq_ (C) for methyl and 0.99 Å, *U*_iso_=1.2*U*_eq_ (C) for methylene.

### Crystal data

**Table:**

C_12_H_14_O_3_	*F*(000) = 440
*M**_r_* = 206.23	*D*_x_ = 1.359 Mg m^−^^3^
Monoclinic, *P*2_1_/*c*	Mo *K*α radiation, λ = 0.71073 Å
Hall symbol: -P 2ybc	Cell parameters from 1569 reflections
*a* = 4.5339 (6) Å	θ = 2.4–25.9°
*b* = 16.815 (2) Å	µ = 0.10 mm^−^^1^
*c* = 13.302 (2) Å	*T* = 89 K
β = 96.495 (8)°	Block, colourless
*V* = 1007.6 (2) Å^3^	0.38 × 0.11 × 0.06 mm
*Z* = 4	

### Data collection

**Table:**

Bruker APEXII CCD area-detector diffractometer	2021 independent reflections
Radiation source: fine-focus sealed tube	1535 reflections with *I* > 2σ(*I*)
graphite	*R*_int_ = 0.063
ω scans	θ_max_ = 26.3°, θ_min_ = 2.9°
Absorption correction: multi-scan (*SADABS*; Bruker 2009)	*h* = −5→5
*T*_min_ = 0.809, *T*_max_ = 1.00	*k* = −20→20
12531 measured reflections	*l* = −13→16

### Refinement

**Table:**

Refinement on *F*^2^	Primary atom site location: structure-invariant direct methods
Least-squares matrix: full	Secondary atom site location: difference Fourier map
*R*[*F*^2^ > 2σ(*F*^2^)] = 0.070	Hydrogen site location: inferred from neighbouring sites
*wR*(*F*^2^) = 0.201	H atoms treated by a mixture of independent and constrained refinement
*S* = 1.13	*w* = 1/[σ^2^(*F*_o_^2^) + (0.0733*P*)^2^ + 1.6783*P*] where *P* = (*F*_o_^2^ + 2*F*_c_^2^)/3
2021 reflections	(Δ/σ)_max_ < 0.001
142 parameters	Δρ_max_ = 0.39 e Å^−^^3^
1 restraint	Δρ_min_ = −0.27 e Å^−^^3^

### Special details

**Table:**

Geometry. All e.s.d.'s (except the e.s.d. in the dihedral angle between two l.s. planes) are estimated using the full covariance matrix. The cell e.s.d.'s are taken into account individually in the estimation of e.s.d.'s in distances, angles and torsion angles; correlations between e.s.d.'s in cell parameters are only used when they are defined by crystal symmetry. An approximate (isotropic) treatment of cell e.s.d.'s is used for estimating e.s.d.'s involving l.s. planes.
Refinement. Refinement of *F*^2^ against ALL reflections. The weighted *R*-factor *wR* and goodness of fit *S* are based on *F*^2^, conventional *R*-factors *R* are based on *F*, with *F* set to zero for negative *F*^2^. The threshold expression of *F*^2^ > σ(*F*^2^) is used only for calculating *R*-factors(gt) *etc*. and is not relevant to the choice of reflections for refinement. *R*-factors based on *F*^2^ are statistically about twice as large as those based on *F*, and *R*- factors based on ALL data will be even larger.

### Fractional atomic coordinates and isotropic or equivalent isotropic displacement parameters (Å^2^)

**Table:**

	*x*	*y*	*z*	*U*_iso_*/*U*_eq_	
O1	0.0748 (4)	0.96722 (11)	0.22522 (15)	0.0219 (5)	
C1	−0.0405 (6)	0.89510 (16)	0.2597 (2)	0.0194 (6)	
C2	−0.2136 (6)	0.90381 (17)	0.3392 (2)	0.0199 (6)	
C21	−0.2918 (7)	0.98596 (17)	0.3742 (2)	0.0269 (7)	
H21A	−0.4854	0.9843	0.4002	0.040*	
H21B	−0.3003	1.0231	0.3171	0.040*	
H21C	−0.1402	1.0037	0.4278	0.040*	
C3	−0.3075 (6)	0.83491 (17)	0.3848 (2)	0.0207 (6)	
C31	−0.4844 (7)	0.83761 (18)	0.4734 (2)	0.0253 (7)	
H31A	−0.4523	0.8888	0.5082	0.038*	
H31B	−0.4208	0.7944	0.5203	0.038*	
H31C	−0.6957	0.8314	0.4496	0.038*	
C4	−0.2276 (6)	0.76058 (17)	0.3479 (2)	0.0207 (6)	
O4	−0.3260 (5)	0.69559 (12)	0.39722 (16)	0.0261 (5)	
H4O	−0.292 (8)	0.6515 (14)	0.369 (2)	0.031*	
C5	−0.0575 (6)	0.75268 (17)	0.2672 (2)	0.0210 (6)	
C51	0.0261 (7)	0.67095 (17)	0.2340 (2)	0.0258 (7)	
H51A	0.1317	0.6425	0.2916	0.039*	
H51B	0.1550	0.6756	0.1799	0.039*	
H51C	−0.1539	0.6415	0.2090	0.039*	
C6	0.0368 (6)	0.82203 (17)	0.2215 (2)	0.0195 (6)	
C7	0.2168 (6)	0.82110 (17)	0.1330 (2)	0.0209 (6)	
H7A	0.1559	0.7753	0.0886	0.025*	
H7B	0.4297	0.8148	0.1578	0.025*	
C8	0.1714 (7)	0.89840 (18)	0.0725 (2)	0.0254 (7)	
H8A	0.3188	0.9010	0.0231	0.031*	
H8B	−0.0288	0.8980	0.0341	0.031*	
C9	0.2022 (6)	0.97048 (17)	0.1380 (2)	0.0224 (6)	
O9	0.3212 (5)	1.03231 (12)	0.11866 (16)	0.0283 (6)	

### Atomic displacement parameters (Å^2^)

**Table:**

	*U*^11^	*U*^22^	*U*^33^	*U*^12^	*U*^13^	*U*^23^
O1	0.0275 (11)	0.0175 (10)	0.0222 (11)	−0.0005 (8)	0.0097 (8)	−0.0005 (8)
C1	0.0225 (14)	0.0163 (14)	0.0189 (14)	−0.0006 (11)	0.0010 (11)	0.0019 (11)
C2	0.0190 (14)	0.0215 (14)	0.0195 (14)	0.0027 (11)	0.0043 (11)	−0.0027 (11)
C21	0.0324 (16)	0.0215 (15)	0.0274 (16)	0.0017 (13)	0.0066 (13)	−0.0017 (12)
C3	0.0212 (14)	0.0230 (14)	0.0180 (14)	0.0017 (11)	0.0024 (11)	0.0004 (11)
C31	0.0302 (16)	0.0233 (15)	0.0241 (16)	0.0018 (12)	0.0100 (13)	−0.0006 (12)
C4	0.0200 (14)	0.0205 (14)	0.0211 (14)	−0.0006 (11)	0.0000 (11)	0.0012 (12)
O4	0.0331 (12)	0.0175 (10)	0.0296 (12)	−0.0004 (9)	0.0117 (9)	0.0031 (9)
C5	0.0230 (15)	0.0193 (15)	0.0210 (15)	0.0009 (11)	0.0033 (12)	0.0008 (11)
C51	0.0297 (16)	0.0200 (15)	0.0289 (16)	0.0031 (12)	0.0087 (13)	0.0007 (12)
C6	0.0172 (13)	0.0224 (14)	0.0188 (14)	0.0020 (11)	0.0020 (11)	0.0002 (11)
C7	0.0212 (14)	0.0206 (14)	0.0214 (15)	0.0012 (11)	0.0047 (12)	−0.0007 (12)
C8	0.0317 (17)	0.0247 (15)	0.0207 (15)	−0.0006 (13)	0.0065 (13)	−0.0012 (12)
C9	0.0231 (14)	0.0231 (15)	0.0214 (15)	0.0024 (12)	0.0042 (12)	0.0027 (12)
O9	0.0360 (12)	0.0206 (11)	0.0300 (12)	−0.0021 (9)	0.0111 (10)	0.0025 (9)

### Geometric parameters (Å, °)

**Table:**

O1—C9	1.354 (3)	C4—C5	1.398 (4)
O1—C1	1.417 (3)	O4—H4O	0.854 (18)
C1—C6	1.390 (4)	C5—C6	1.404 (4)
C1—C2	1.394 (4)	C5—C51	1.505 (4)
C2—C3	1.397 (4)	C51—H51A	0.9800
C2—C21	1.512 (4)	C51—H51B	0.9800
C21—H21A	0.9800	C51—H51C	0.9800
C21—H21B	0.9800	C6—C7	1.506 (4)
C21—H21C	0.9800	C7—C8	1.530 (4)
C3—C4	1.405 (4)	C7—H7A	0.9900
C3—C31	1.500 (4)	C7—H7B	0.9900
C31—H31A	0.9800	C8—C9	1.490 (4)
C31—H31B	0.9800	C8—H8A	0.9900
C31—H31C	0.9800	C8—H8B	0.9900
C4—O4	1.374 (3)	C9—O9	1.212 (4)
			
C9—O1—C1	121.4 (2)	C4—C5—C51	119.4 (3)
C6—C1—C2	123.9 (3)	C6—C5—C51	122.2 (3)
C6—C1—O1	121.3 (2)	C5—C51—H51A	109.5
C2—C1—O1	114.6 (2)	C5—C51—H51B	109.5
C1—C2—C3	117.9 (3)	H51A—C51—H51B	109.5
C1—C2—C21	120.1 (3)	C5—C51—H51C	109.5
C3—C2—C21	122.0 (3)	H51A—C51—H51C	109.5
C2—C21—H21A	109.5	H51B—C51—H51C	109.5
C2—C21—H21B	109.5	C1—C6—C5	118.3 (3)
H21A—C21—H21B	109.5	C1—C6—C7	118.5 (3)
C2—C21—H21C	109.5	C5—C6—C7	123.3 (2)
H21A—C21—H21C	109.5	C6—C7—C8	110.5 (2)
H21B—C21—H21C	109.5	C6—C7—H7A	109.6
C2—C3—C4	118.9 (3)	C8—C7—H7A	109.6
C2—C3—C31	122.2 (3)	C6—C7—H7B	109.6
C4—C3—C31	118.9 (3)	C8—C7—H7B	109.6
C3—C31—H31A	109.5	H7A—C7—H7B	108.1
C3—C31—H31B	109.5	C9—C8—C7	112.6 (2)
H31A—C31—H31B	109.5	C9—C8—H8A	109.1
C3—C31—H31C	109.5	C7—C8—H8A	109.1
H31A—C31—H31C	109.5	C9—C8—H8B	109.1
H31B—C31—H31C	109.5	C7—C8—H8B	109.1
O4—C4—C5	121.9 (2)	H8A—C8—H8B	107.8
O4—C4—C3	115.5 (2)	O9—C9—O1	117.4 (3)
C5—C4—C3	122.6 (3)	O9—C9—C8	126.0 (3)
C4—O4—H4O	113 (2)	O1—C9—C8	116.6 (2)
C4—C5—C6	118.4 (3)		

### Hydrogen-bond geometry (Å, °)

**Table:**

Cg2 is the centroid of the C1–C6 benzene ring.

**Table:**

*D*—H···*A*	*D*—H	H···*A*	*D*···*A*	*D*—H···*A*
O4—H4O···O9^i^	0.85 (2)	2.02 (3)	2.754 (3)	144 (3)
C8—H8B···O9^ii^	0.99	2.58	3.395 (4)	140
C8—H8B···O4^iii^	0.99	2.66	3.440 (4)	136
C7—H7B···Cg2^iv^	0.99	2.61	3.505 (3)	150
C31—H31C···Cg2^v^	0.98	2.62	3.512 (3)	151

Symmetry codes: (i) −*x*, *y*−1/2, −*z*+1/2; (ii) −*x*, −*y*+2, −*z*; (iii) *x*, −*y*+3/2, *z*−1/2; (iv) *x*−1, *y*, *z*; (v) *x*+1, *y*, *z*.
